# Island restoration to rebuild seabird populations and amplify coral reef functioning

**DOI:** 10.1111/cobi.14313

**Published:** 2024-06-18

**Authors:** Ruth E. Dunn, Cassandra E. Benkwitt, Olivier Maury, Nicolas Barrier, Peter Carr, Nicholas A. J. Graham

**Affiliations:** ^1^ Lancaster Environment Centre Lancaster University Lancaster UK; ^2^ The Lyell Centre Heriot‐Watt University Edinburgh UK; ^3^ Institut de Recherche pour le Développement Université de Montpellier Sète France; ^4^ Institute of Zoology Zoological Society of London London UK

**Keywords:** cross‐ecosystem nutrients, ecological process, energetics, habitat restoration, invasive species, resilience, tropics, energéticos, especie invasora, nutrientes transecosistémicos, procesos ecológicos, resiliencia, restauración de hábitat, trópicos, 跨生态系统的营养物质, 入侵物种, 生态过程, 能量学, 恢复力, 栖息地恢复, 热带

## Abstract

Mobile organisms like seabirds can provide important nutrient flows between ecosystems, but this connectivity has been interrupted by the degradation of island ecosystems. Island restoration (via invasive species eradications and the restoration of native vegetation) can reestablish seabird populations and their nutrient transfers between their foraging areas, breeding colonies, and adjacent nearshore habitats. Its diverse benefits are making island restoration increasingly common and scalable to larger islands and whole archipelagos. We identified the factors that influence breeding seabird abundances throughout the Chagos Archipelago in the Indian Ocean and conducted predictive modeling to estimate the abundances of seabirds that the archipelago could support under invasive predator eradication and native vegetation restoration scenarios. We explored whether the prey base exists to support restored seabird populations across the archipelago, calculated the nitrogen that restored populations of seabirds might produce via their guano, and modeled the cascading conservation gains that island restoration could provide. Restoration was predicted to increase breeding pairs of seabirds to over 280,000, and prey was predicted to be ample to support the revived seabird populations. Restored nutrient fluxes were predicted to result in increases in coral growth rates, reef fish biomasses, and parrotfish grazing and bioerosion rates. Given these potential cross‐ecosystem benefits, our results support island restoration as a conservation priority that could enhance resilience to climatic change effects, such as sea‐level rise and coral bleaching. We encourage the incorporation of our estimates of cross‐ecosystem benefits in prioritization exercises for island restoration.

## Abstract (es)

Restauración en islas para reconstruir las poblaciones de aves marinas y amplificar la funcionalidad de los arrecifes de coral

**Resumen:**

Los organismos móviles como las aves marinas pueden proporcionar flujos importantes de nutrientes entre los ecosistemas, aunque esta conectividad ha sido interrumpida por la degradación de los ecosistemas isleñas. La restauración de islas (por medio de la erradicación de especies invasoras y la restauración de la vegetación nativa) puede reestablecer las poblaciones de aves marinas y su transferencia de nutrientes entre las áreas de forrajeo, las colonias reproductoras y los hábitats adyacentes a la costa. Los diferentes beneficios de la restauración de islas hacen que sea cada vez más común y escalable a islas más grandes y archipiélagos completos. Identificamos los factores que influyen sobre la abundancia de aves reproductoras en todo el archipiélago de Chagos en el Océano Índico y realizamos un modelo predictivo para estimar la abundancia de aves que podría soportar el archipiélago bajo escenarios de la erradicación de un depredador invasor y la restauración de la vegetación nativa. Exploramos si existe la base de presas para soportar las poblaciones restauradas de aves marinas en el archipiélago, calculamos el nitrógeno que las poblaciones restauradas podrían producir mediante el guano y modelamos la conservación en cascada que podría proporcionar la restauración de la isla. Se pronosticó que la restauración incrementaría las parejas reproductoras a más de 280,000 y que las presas serían las suficientes para soportar las poblaciones restauradas de aves marinas. También se pronosticó que los flujos restaurados de nutrientes resultarían en un incremento de la tasa de crecimiento de los corales, la biomasa de los peces del arrecife y las tasas de bio‐erosión y de alimentación de los peces loro. Dados estos beneficios potenciales entre los ecosistemas, nuestros resultados respaldan a la restauración de islas como una prioridad de conservación que podría incrementar la resiliencia a los efectos del cambio climático, como el incremento en el nivel del mar y el blanqueamiento de los corales. Promovemos que se incorporen nuestras estimaciones de los beneficios transecosistémicos dentro de los ejercicios de priorización para la restauración de islas.

## 【摘要】

海鸟等移动的生物可在生态系统之间提供重要的营养物质流, 但生态系统退化会破坏这种连通性。岛屿恢复(通过消灭入侵物种和恢复原生植被)可以重建海鸟种群, 并在其觅食区、繁殖地和邻近的近岸栖息地之间进行营养物质转移。种种益处使得海岛恢复正变得越来越普遍, 并可扩展到更大的岛屿和整个群岛。本研究确定了影响印度洋查戈斯群岛海鸟繁殖数量的因素, 并通过预测建模估计了在清除入侵捕食者和恢复原生植被的情况下, 该群岛可支持的海鸟数量。我们进一步探究了整个群岛是否存在充足猎物来支持恢复后的海鸟种群, 估算了恢复后种群通过鸟粪产生的氮, 并模拟了岛屿恢复可能提供的级联保护。据预测, 岛屿恢复后海鸟繁殖对数量将增加到28万以上, 猎物也将足以支持恢复后的海鸟种群。营养物质流动的恢复预计还将导致珊瑚生长率、珊瑚鱼生物量以及鹦嘴鱼啃食和生物侵蚀率的增加。鉴于这些潜在的跨生态系统效益, 我们的研究结果支持将岛屿恢复作为优先保护事项, 以增强抵抗气候变化影响(如海平面上升和珊瑚白化)的恢复力。我们鼓励在岛屿恢复的优先保护实践中纳入本研究对跨生态系统效益的估计。**【翻译: 胡怡思; 审校: 聂永刚】**

## INTRODUCTION

Though oceanic islands comprise only ∼5% of Earth's land area, they host disproportionate densities of plant and animal species diversity (Tershy et al., 2015). Their geographic isolation means that island assemblages are characterized by range‐restricted species and elevated rates of endemism (Kier et al., 2009). These highly adapted island taxa can exhibit slow life‐history strategies and typically have reduced predatory defenses, thereby heightening their susceptibility to threats (Anton et al., 2020). Furthermore, human activity on islands has led to habitat loss and introductions of non‐native invasive species (hereafter invasive species) that are linked to biodiversity losses (Kier et al., 2009; Spatz et al., 2017). Exacerbating these threats, island landmasses are inherently vulnerable to perturbations due to their isolation, often low‐lying geomorphology, and exposure to sea‐level rise and storms that are likely to increase in severity and frequency with climate change (Wetzel et al., 2013). Islands are therefore epicenters of extinction, representing 80% of historic species losses (Ricketts et al., 2005), likely triggering reductions in ecosystem connectivity (Berti & Svenning, 2020). This means that islands also represent a unique conservation opportunity: the effectiveness of island restoration efforts, the preconditions that maximize its effectiveness, and the cross‐ecosystem outcomes of such interventions have become important research frontiers (Holmes et al., 2019).

Seabirds are one of the most threatened taxa (Croxall et al., 2012), yet they are integral components of insular ecosystems (Grant et al., 2022). They congregate at terrestrial breeding colonies, and in the absence of threats to their populations, the size and distribution of their breeding colonies are largely driven by the availability of sufficient prey in nearby waters (Ashmole, 1963). Through their nesting activities, seabirds generate physical disturbance, influencing plant biomass, species richness, and community composition (Ellis, 2005). Although they nest on land, most seabird species forage for prey across coastal and pelagic ecosystems, acting as “mobile link organisms” (Lundberg & Moberg, 2003) and exerting chemical influence among their terrestrial breeding grounds via the transfer of nitrogen‐ and phosphorus‐rich oceanic nutrients from marine foraging habitats (Grant et al., 2022). Although tropical waters are considered nutrient‐poor environments, nutrient‐rich guano fertilizer permeates throughout seabird colonies and into adjacent nearshore ecosystems, leading to increased nutrient uptake, fish biomass, and rates of ecosystem functioning (Graham et al., 2018). Furthermore, guano‐derived nitrogen and phosphorus can be assimilated into coral and zooxanthellae tissues, contributing to their nitrogen requirements and enhancing their growth (Lorrain et al., 2017; Savage, 2019).

Seabirds face a multitude of threats, including global climate change and the industrialization of fishing practices, that have consequences, including threats of bycatch and changes in the distribution, availability, and accessibility of prey, with subsequent implications for seabird population dynamics (Barbraud et al., 2018). In addition to at‐sea threats, the presence of invasive species in their breeding habitats threatens seabirds globally, detrimentally affecting the population trajectories of 46% of seabird species and over 170 million individuals (Dias et al., 2019; Spatz et al., 2023). For example, invasive rats are estimated to be present across 90% of the world's archipelagos (Atkinson, 1985), where they directly prey on seabird eggs, chicks, and adults (Jones et al., 2008). Furthermore, land‐use changes, such as human‐facilitated expansions of trees and crop plants (e.g., coconut palm [*Cocos nucifera*]), have led to reduced seabird densities due to unfavorable nesting habitats (McCauley et al., 2012). The integral role of seabirds among interconnected island and marine ecosystems means that the detrimental impacts of industrial fisheries, invasive species, and habitat loss transcend ecosystem boundaries and trophic levels (TLs) (Grant et al., 2022). For example, in addition to seabird population declines, changes in fishing practices and, subsequently, changes in forage fish stocks can cause seabirds to feed on alternative prey or in novel environments (Bicknell et al., 2013). Furthermore, invasive species have caused breakages in chains of interactions among seabird fertilization effects, soil nutrients, and native plant growth (Wardle et al., 2012), as well as coastal nutrients, plankton abundances, and the occurrence of manta ray (*Manta birostris*) aggregations (McCauley et al., 2012).

Fortunately, the eradication of invasive species from islands is a proven and increasingly common conservation tool, undertaken with goals for reversing seabird population declines and biodiversity losses (Spatz et al., 2022). Invasive mammal eradications have now been attempted on over 1000 islands and, in combination with other restoration actions, such as native species translocations, have aided the recovery of hundreds of native island taxa worldwide (DIISE, 2018; Jones et al., 2016). Recently, downstream ecological effects of these conservation actions have been documented. For example, predator eradications on islands in New Zealand led to restored seabird populations, deposition of nutrient‐rich guano, fertilized nearshore marine habitats, and increased macroalgal diversity (Rankin & Jones, 2021). Furthermore, coral reef communities surrounding islands without rats in the Indian Ocean have higher nitrogen isotope values, faster fish growth rates, and differing benthic community structure in comparison with those where rats have decimated seabird populations (Benkwitt et al., 2019; Benkwitt, Taylor, et al., 2021; Graham et al., 2018). The benefits of eradications can therefore be broad in scope, not only facilitating the restoration of terrestrial island biodiversity but also propagating throughout ecosystem linkages via nutrient flows and trophic relationships (Jones, 2010b). Indeed, signals of rebuilt cross‐ecosystem linkages following invasive species eradications and seabird population recoveries (determined via the nutrient signatures of marine tissues) are evident over 1 km from shore (Benkwitt, Gunn, et al., 2021).

Currently, much island restoration work is conducted under the assumption that if terrestrial breeding habitats are restored, seabirds will return, potentially aided by chick translocations and the encouragement of acoustic and visual stimuli, such as playbacks and decoys (Jones & Kress, 2012; VanderWerf et al., 2023). However, despite their importance, predator–prey dynamics in seabird foraging grounds are rarely considered in restoration efforts, likely due to most island management projects having a narrower focus than the restoration of whole communities or ecosystems (Jones et al., 2011). At the same time that island ecosystems have suffered degradation, climate change and extraction via fisheries have also affected seabird prey availability (Durrett & Mulder, 2011), threatening the forage fish biomass required to sustain seabird productivity (Cury et al., 2011). Therefore, estimates of pelagic prey populations in the foraging ranges of seabirds should be estimated to establish whether the marine ecosystems that surround restored islands can support large seabird population increases.

We sought to investigate the capacity of a protected marine prey base to support the energy requirements of restored seabird populations and the scale of opportunity for restoring seabird‐derived nutrient subsidies to benefit coral reefs across a tropical island archipelago. To do this, we assessed the potential benefits of island restoration actions, namely, invasive species eradications and the restoration of native vegetation, as means to restore tropical seabird populations and coral reef functioning in the Chagos Archipelago. The Chagos Archipelago, situated in a large marine protected area in the central Indian Ocean, is composed of ∼55 islands across 5 islanded coral reef atolls (Sheppard, 2016). Although 4 of these atolls are currently uninhabited (the fifth, Diego Garcia, contains a military facility), black rats *Rattus rattus* and coconut palm forest were introduced in the late 18th and early 19th centuries and are present on many of the islands (Sheppard, 2016). Of these atoll islands, although many are degraded, 24 are rat free and host large seabird populations (Carr et al., 2021). Thereby, they form a large‐scale natural experiment through which to investigate the influence of human‐introduced mammalian predators and coconut palm on atoll island and adjacent marine ecosystems. First, we identified the factors that influence breeding seabird abundances throughout the Chagos Archipelago and used our resultant models to predict the abundances of seabirds that the archipelago could support under 3 rat eradication and native vegetation restoration scenarios. We then calculated the energy requirements of the archipelago's seabird populations under these scenarios and compared prey requirement estimates with size‐structured bioenergetic models of epipelagic fish availability in the foraging ranges of the seabirds. Finally, we calculated the nitrogen that restored populations of seabirds might produce via their guano and modeled the expected influence of restored nutrient fluxes on coral growth, reef fish biomass, parrotfish grazing, and parrotfish bioerosion.

## METHODS

### Influence of island restoration on seabird populations

We identified the terrestrial factors that influenced the abundance of 3 key seabird species across the Chagos Archipelago (5° 50′ S, 72° 00′ E) and used the resultant models to predict their potential population sizes across the Archipelago following rat eradications and native vegetation restoration. The Chagos Archipelago hosts over 280,000 pairs of breeding seabirds, and 96% of this assemblage is composed of lesser noddies (*Anous tenuirostris*), sooty terns (*Onychoprion fuscatus*), and red‐footed boobies (*Sula sula*) (Carr et al., 2020); thus, we focused on these species for our analyses. These species represent populus shrub‐, ground‐, and tree‐nesting taxa, respectively, that represent a range of foraging strategies (Benkwitt et al., 2022). To identify the factors influencing the population sizes of these species, we compared their abundances (breeding pairs), derived from previously published seabird censuses conducted from 2008 to 2018 across 25 atoll islands (those for which data on area and habitat cover were available [Appendix S1]).

Seabird abundance data were zero‐inflated to a level that could not be accommodated by common count distributions (e.g., Poisson or negative binomial), contained some very high values, and did not have a common average (Appendix S2); therefore, we used a Bayesian hurdle lognormal regression model (Feng, 2021). This model was a mixture of 2 processes, dependent on whether seabird abundance values were larger than zero, as influenced by rat status (present or absent). We included rat status, island area, and proportion of native (not coconut palm) forest, savannah, and wetland land cover (mean = 43%, range = 3–100%) as explanatory variables for when seabird abundances were >0 and used the priors outlined in Appendix S3. We used species‐specific intercepts and incorporated island as a random intercept to help account for spatial nonindependence. We used the log transformation on island area to reduce skew and ensure sufficient generalization of the data to make valid predictions. We confirmed model convergence via visually inspecting the resultant Markov chain Monte Carlo (MCMC) method chains and calculating a Gelman–Rubin convergence statistic ($\hat{R}$) of 1 (McElreath, 2020). We validated our model with posterior predictive check plots to compare our seabird abundance data with simulated data from the posterior predictive distribution. Past records of seabird abundances throughout the Chagos Archipelago were not available, so we assessed the models’ predictive ability with leave‐one‐out cross‐validation to estimate the expected log predictive density of a new data set (Gabry, 2023). Pareto *k* estimates revealed that the model predicted 100% of observations with acceptable accuracy (*k* < 0.7) and 94.7% with high accuracy (*k* < 0.5).

We used the log‐normal regression component of our model of the factors influencing breeding lesser noddy, sooty tern, and red‐footed booby abundances in the fitted.brms function (Bürkner, 2017). This function allowed us to estimate the populations of these species that could inhabit 25 currently rat‐infested atoll islands of known area in the Chagos Archipelago if the rats were eradicated and native vegetation cover was restored under 3 different restoration scenarios. To make these predictions, we used data on the islands’ areas and assumed one of 3 restoration scenarios would reflect native vegetation being unrestored or restored to varying degrees: rats eradicated and presence of 25% native vegetation cover (approximately the lower quartile of the proportion of native vegetation cover across the islands of the Chagos Archipelago); rats eradicated and native vegetation cover restored to 50% (approximately the mean of the proportion of native vegetation cover across the archipelago); and rats eradicated and native vegetation cover restored to 75% (approximately the upper quartile of the proportion of native vegetation cover across the archipelago). We excluded the island of Diego Garcia because of the potential confounding influence of its human population.

### Prey requirements of seabird populations

**FIGURE 1:**
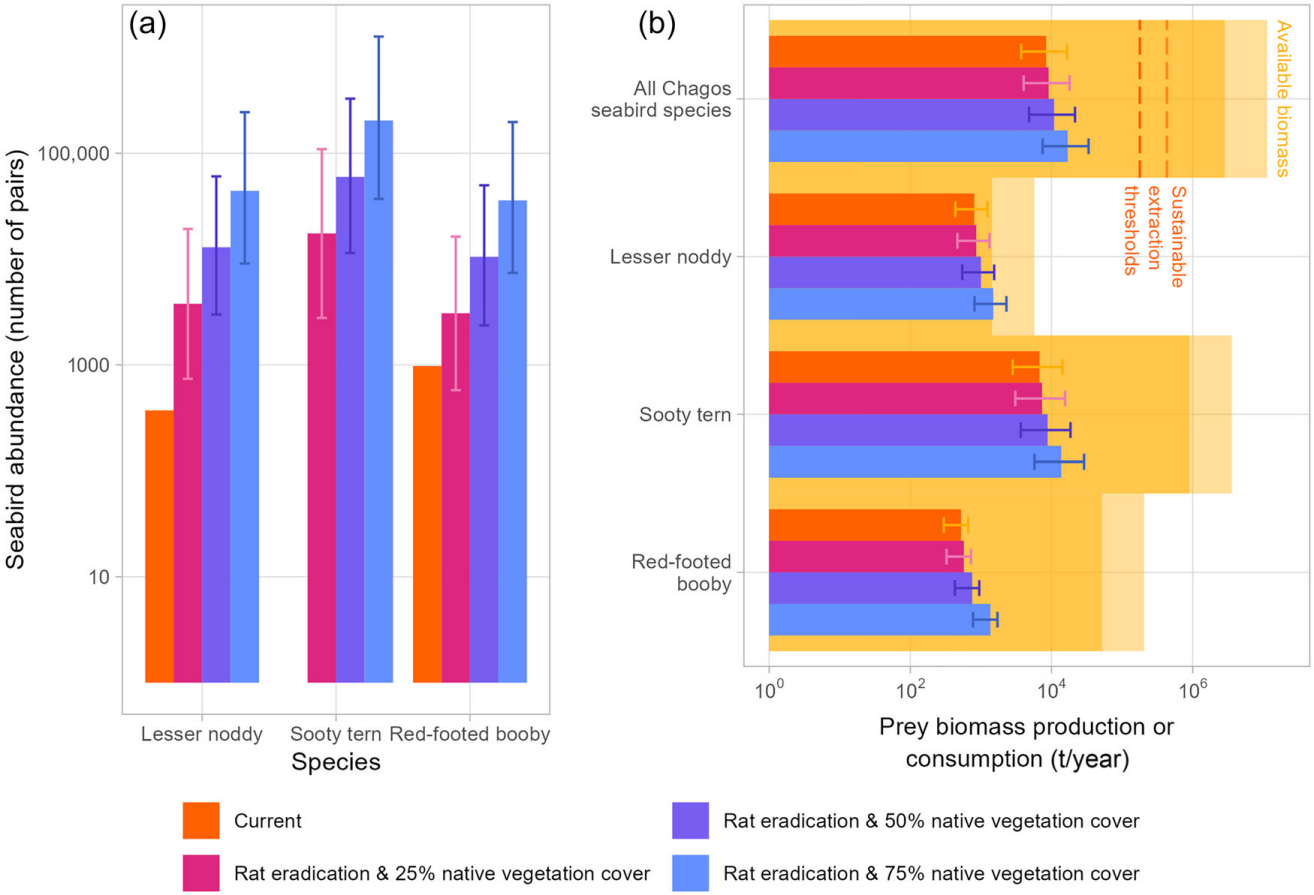
Abundances and prey requirements of lesser noddies (*Anous tenuirostris*), sooty terns (*Onychoprion fuscatus*) (currently zero breeding pairs), and red‐footed boobies (*Sula sula*) throughout the Chagos Archipelago under current conditions and under 3 rat eradication and native habitat restoration scenarios: (a) current total abundance of breeding pairs across 25 currently rat‐infested atoll islands (orange) and predicted abundances under 3 restoration scenarios (log scale; current abundances, count data with no associated error; height of bars, posterior mean model predictions; error bars, quantile‐based 95% intervals) and (b) epipelagic prey biomass production in the maximum breeding foraging ranges of all seabird species breeding on the archipelago (log scale; yellow, the darker the shading, the lower the biomass production) relative to consumption requirements of all the archipelago's seabirds and that of the 3 focus species (log scale; orange, current consumption requirements; pink, purple, and blue, predictions of seabird consumption requirements under restoration scenarios; height of bars, posterior mean; error bars, upper and lower Bayesian credible intervals; orange dashed lines, sustainable extraction threshold [beyond which the biomass consumed by seabirds threatens ecosystem]; dark line, low range of threshold; light line, high range of threshold).

We investigated the propensity for the offshore marine environment around the Chagos Archipelago to support the epipelagic prey consumption requirements of the Archipelago's current seabird populations and those predicted for lesser noddies, sooty terns, and red‐footed boobies under the 3 rat eradication and native vegetation restoration scenarios. We simulated the availability of seabird prey biomass with the Apex Predators ECOSystem Model (APECOSM) (Maury, 2010; Maury & Poggiale, 2013; Maury et al., 2007). The APECOSM is a theoretical mechanistic size‐structured bioenergetic model that simulates the 3‐dimensional dynamics of marine organisms, such as fishes and cephalopods, based on dynamic energy budget theory (Kooijman, 2000). From this model, due to the foraging ecology of the tropical seabird species (Ballance & Pitman, 1999), we extracted APECOSM outputs of the monthly daytime biomass of epipelagic forage fish (3–20 cm total length) present in the top 20 m of the water column of the ocean surrounding the Chagos Archipelago (15°S–2°N; 60°E–82°E). These cutoffs corresponded with the gape limits and maximum foraging depths of the Archipelago's seabird assemblage, some species of which dive up to 60 m below the surface to capture prey (Burger, 2001; Surman & Wooller, 2003). To calculate the biomass of prey available to all the seabird species in the Chagos Archipelago, we extracted the mean annual forage fish biomass from 2008 to 2018 from a 1200‐km‐radius area that encompassed the maximum breeding foraging ranges of all the Archipelago's seabirds (Graham et al., 2018). We then performed prey biomass extractions from the maximum foraging ranges of our 3 focal species: lesser noddies breeding in Western Australia in the eastern Indian Ocean (110 km) (Surman et al., 2017), sooty terns breeding in the Seychelles in the western Indian Ocean (890 km) (Neumann et al., 2018), and red‐footed boobies breeding in the Chagos Archipelago (400 km) (Trevail & Wood et al., 2023). As a comparison, to evaluate the prey available to seabirds if they did not travel so far, we also extracted prey biomass from the mean foraging ranges of these 3 species: 36, 310, and 110 km, respectively (Neumann et al., 2018; Surman et al., 2017; Trevail & Wood et al., 2023). To calculate prey biomass production, we multiplied our prey biomass estimates by production‐to‐biomass ratios (*P*/*B*) or annual turnover rates, considering trophic level (TL) effects. Prey TL was assumed to be from 3.0 to 3.7 based on representative prey species (*Exocoetus volitans* [blue flying fish] TL = 3.0 [SE 0.09]; *Parupeneus chrysonemus* [yellow‐threaded goatfish] TL = 3.4 [0.4]; *Cheilopogon atrisignis* [glider flying fish] TL = 3.7 [0.5] [Froese & Pauly, 2022]). Global *P*/*B* ratios for these TLs were assumed to be 3.4 and 1.4 per year for TL 3.0 and 3.7, respectively, based on the relationship between *P*/*B* ratios and mean TL across 110 Ecopath models in Kolding et al. (2016) (*R* ^2^ = 0.92). For their consumption to be sustainable, seabird extraction rates of forage fish should not exceed 25% of biomass production (Smith et al., 2011), so we used this criterion as a threshold beyond which the biomass extracted by all the Archipelago's seabirds could threaten the ecosystem via the overexploitation of forage fishes. We therefore assumed that, while remaining ecologically sustainable, the Chagos Archipelago's entire seabird population could extract a maximum of 0.35–0.85 times prey biomass per year (25% of 1.4 and 3.4, respectively) (Figure 1b).

We compared our estimates of seabird prey production with the current annual consumption requirements of the breeding seabirds of the Chagos Archipelago and those predicted under the 3 restoration scenarios (assuming a change in the consumption requirements of only the 3 focal species). We obtained species‐specific body mass values (Schreiber & Burger, 2001) and used these in the Seabird FMR Calculator (Dunn et al., 2018) to estimate field metabolic rates that were species, latitude, and breeding‐phase specific. We combined field metabolic rate estimates with prey energy densities (5.5 kJ/g [Clarke & Prince, 1980]) and assimilation rates (75% [Jackson, 1986]) to obtain the daily biomass consumption of the Chagos Archipelago's breeding seabirds. Annual seabird consumption across the entire Chagos Archipelago was therefore calculated as the product of our species‐specific daily biomass consumption estimates, current and predicted seabird population sizes (Carr et al., 2020), and length of the species’ breeding seasons (Schreiber & Burger, 2001). The breeding phenology of these species is not fully understood throughout the Chagos Archipelago, but it is likely that most species forage farther from their breeding colonies during their nonbreeding seasons (Trevail et al., 2023). We compared the production of seabird prey biomass in the foraging areas of the Chagos Archipelago's seabirds with their current and predicted consumption requirements.

### Influence of restored seabird populations on coral reefs

For each island, we calculated annual seabird nitrogen input for the populations of seabirds that currently inhabit the Chagos Archipelago (encompassing all breeding seabird species; Carr et al., 2021), as well as for the populations predicted under the 3 restoration scenarios (featuring increases to the lesser noddy, sooty tern, and red‐footed booby population sizes only). We followed preestablished methods (Carr et al., 2021; Schreiber & Burger, 2001; Young et al., 2010); thereby, we used species‐specific mass values and allometric scaling to estimate seabird defecation rates and summed these with the nitrogen content of guano, the abundance of each species (both current and predicted), and the length of their breeding season in days (Carr et al., 2021; Schreiber & Burger, 2001; Young et al., 2010).

We predicted the influence of increased seabird‐derived nitrogen on the coral reef systems adjacent to the islands of the Chagos Archipelago and applied these predictions to calculate the potential for coral reef metrics (i.e., coral growth, reef fish biomasses, parrotfish grazing, and parrotfish bioerosion) to increase throughout the entire Archipelago. To do this, we measured coral growth. Small branching *Acropora* colonies within 300 m of the shore were tagged for identification, photographed alongside a scale bar, and then revisited and rephotographed a year later (in either 2019, 2020, or 2021) so that change in planar area over time could be calculated (methodological details in Benkwitt et al. [2023]). Coral growth rates were calculated for shallow, lagoonal reef areas adjacent to 8 islands in the Chagos Archipelago (4 where rats were present and 4 where they were absent). Next, reef fish biomass was evaluated in 2015 through 4 replicate underwater transect surveys (each 30 × 5 m) conducted along the reef crests of islands (methodological details in Graham et al. [2018]). Finally, rates of parrotfish grazing and bioerosion were calculated based on reef fish densities and size‐ and species‐specific consumption rates (as in Graham et al. [2018]). Reef fish metrics were calculated at 12 islands (6 where rats were present and 6 where they were absent). Fieldwork was undertaken under permit numbers 0005SE15, 0004SE18, 0001SE19, 0003SE20, and 0002SE21.

We created a set of lognormal regression models to make predictions of the influence of seabird‐derived nitrogen input on coral growth, reef fish biomass, parrotfish grazing, and parrotfish bioerosion, incorporating island as a random intercept in these models. As before, model convergence was confirmed via visual inspections of the resultant MCMC chains and $\hat{R}$, model validation was assessed via posterior predictive check plots, and the models’ predictive ability was assessed using leave‐one‐out cross‐validation. Pareto *k* estimates revealed that all 4 models predicted 100% of observations with acceptable accuracy and > 91% with high accuracy. We used these models in the fitted.brms function to make predictions of coral reef metrics across all islands throughout the Chagos Archipelago (other than Diego Garcia) under the 3 restoration scenarios, based on the models’ posterior predictive distributions. Furthermore, we used the prediction results to calculate the potential reef fish biomass that could be supported across the entire 4380 km^2^ of shallow (<20‐m deep) coral reef area in the Chagos Archipelago (Sheppard et al., 2012).

All analyses were run in R (R Core Team, 2022) and implemented in Stan (Stan Development Team, 2022) with the brms package (Bürkner, 2017). For all models, unless otherwise stated, we used the default brms priors and ran the model with 4 MCMC chains for 3000 iterations and a warmup of 1000 iterations.

### Data availability

Chagos Archipelago vegetation data are publicly available via the Chagos Information Portal (https://chagosinformationportal.org/), and seabird population data are publicly available in the online supporting information of Carr et al. (2020). Data on fish biomass and erosion and grazing rates are publicly available on GitHub (https://github.com/mamacneil/ChagosRats) as are coral growth rate data (https://github.com/cbenkwitt/seabirds‐coral‐reef‐recovery) and all code relevant to this article (https://github.com/RuthDunn/Seabird_nutrients_coral_potential).

## RESULTS

### Influence of island restoration on seabird populations

Rat presence had a negative effect on lesser noddy, sooty tern, and red‐footed booby breeding abundances. Islands that had rats had a higher probability of hosting 0 pairs of seabirds than island without rats (standardized estimates = 1.42, 95% highest posterior density [HPD]: 0.74–2.10) (Appendix S4). Islands where rats were present (excluding Diego Garcia) currently had no breeding sooty terns, 370 red‐footed booby pairs, and 974 lesser noddy pairs (Figure 1a). Atoll island size and native vegetation cover had positive influences on the abundances of breeding lesser noddies, sooty terns, and red‐footed boobies across the Chagos Archipelago (Appendix S4). Seabird abundances were higher on large islands where there was a higher proportion of native vegetation cover (including native forest, savannah, and wetland habitats) than on small islands with coconut palm.

When we predicted the potential for if rats were eradicated from 25 of the currently rat‐infested islands (those where island area and vegetation cover data were available; ∼45% of the Chagos Archipelago's islands), we predicted that populations of lesser noddies, sooty terns, and red‐footed boobies across these islands could increase 18‐fold. The islands had the potential to support a total of ∼3800 (HPD: 740–19,000), 17,000 (HPD: 2700–110,000), and 3100 (HPD: 570–16,000) breeding pairs of lesser noddies, sooty terns, and red‐footed boobies, respectively (Figure 1a). The restoration of 50% native habitat on the islands modeled for rat eradication could lead to a further 3‐fold increase in seabird populations to a total of 83,000 pairs (HPD: 17,000–438,000) (Figure 1a), whereas 75% native habitat could lead to a total of 280,000 pairs on currently rat‐infested islands (HPD: 54,000–1,200,000) (Figure 1a).

### Prey requirements of seabird populations

We estimated that the Chagos Archipelago's entire current seabird population consumed 8300 tons of prey annually from the pelagic environment (Bayesian credible interval [CRI]: 3700–17,000) (Figure 1b). Of this, sooty terns consumed the majority (6700 tons/year, CRI: 2800–14,000), lesser noddies consumed 800 tons/year (CRI: 440–1200), and red‐footed boobies consumed 520 tons/year (CRI: 300–660).

Despite high consumption levels, our size‐based model estimates suggested that there was generally adequate epipelagic prey available in the offshore waters around the Chagos Archipelago to sustainably support the energy requirements of restored populations of lesser noddy, sooty tern, and red‐footed booby populations following rat eradication and native vegetation restoration (prey production ranged from mean [SD] 2,800,000 tons/year [1,000,000] to 11,000,000 tons/year [4,000,000], depending on rates of biomass production) (Figure 1b). Estimates of epipelagic prey consumption by all seabird species (current populations and those predicted for lesser noddies, sooty terns, and red‐footed boobies under rat eradication and native vegetation restoration scenarios) were below the corresponding threshold for ecologically sustainable extraction levels (180,000–440,000 tons/year) (Figure 1b). Even when accounting for overlap in seabird species’ foraging ranges, the differences in their consumption requirements and the available prey were great (Figure 1b), and only the consumption requirements of the lesser noddy populations predicted under the restoration scenario where rats were eradicated and 75% of native vegetation cover was restored risked being higher than the available prey biomass (consumption 1500 tons/year, CRI: 810–2300; lower prey biomass production estimate = 1400 tons/year [780]).

### Influence of restored seabird populations on coral reefs

Currently, 78 tons/year of seabird‐derived nitrogen is deposited on the Chagos Archipelago's atoll islands. We estimated that restored seabird populations have the potential to produce 84 tons/year if rats were eradicated and there was 25% native vegetation cover, 104 tons/year if 50% native vegetation cover was restored and rats were eradicated, and 170 tons/year if 75% native vegetation cover was restored and rats were eradicated.

**FIGURE 2:**
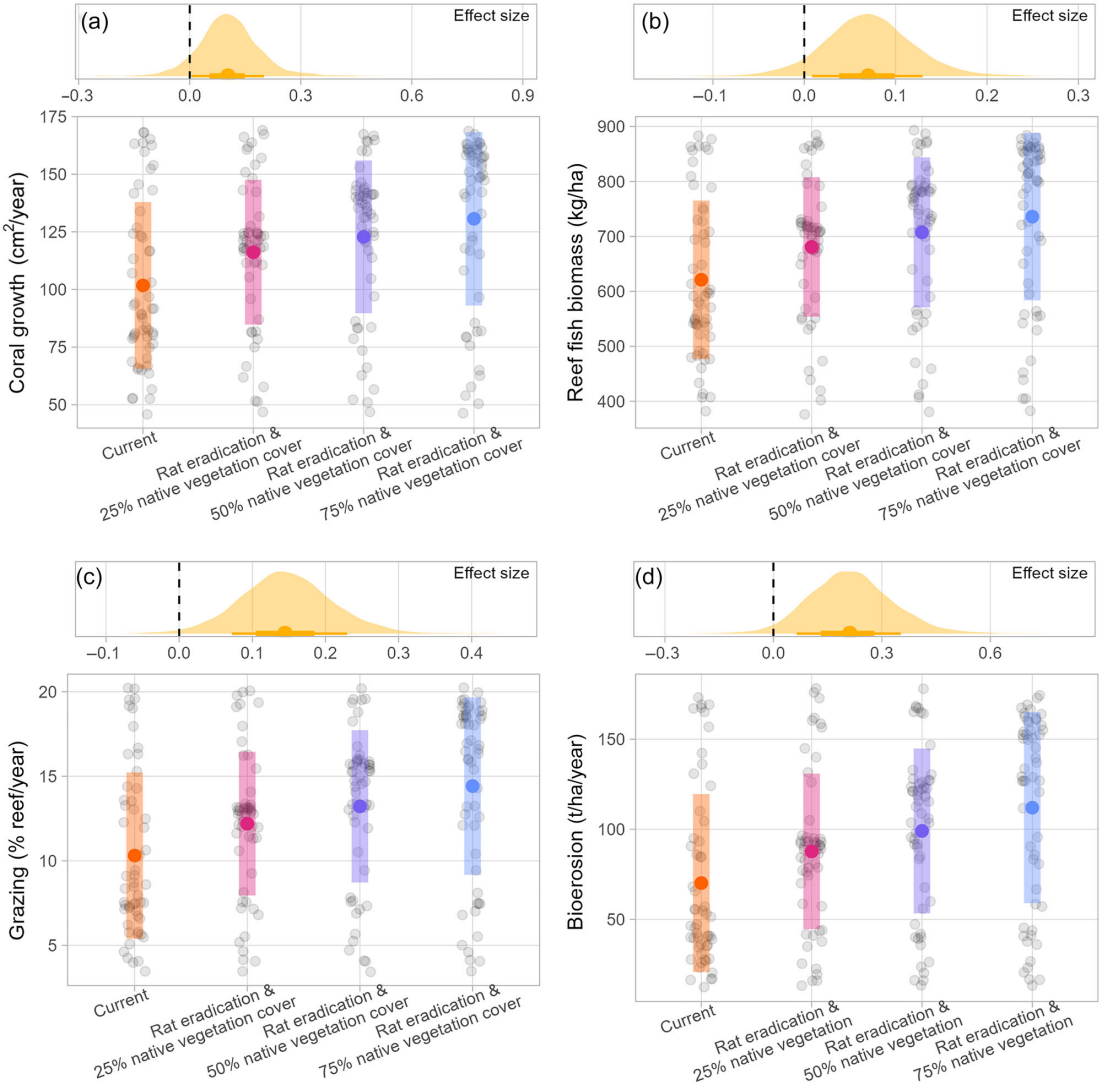
Rates of (a) coral growth, (b) reef fish biomass, (c) parrotfish grazing, and (d) parrotfish bioerosion under current conditions (orange) and as predicted under rat eradication and habitat restoration scenarios (pink, purple, blue) and highest posterior density regions of the effect size for the log‐linear effect of seabird‐derived nitrogen input (kg·ha^−1^·year ^−1^) (small graphs) across 52 atoll islands in the Chagos Archipelago, 22 of which are currently rat‐infested (gray points, mean prediction for each atoll island; colored points, overall mean; bars, standard deviation).

We observed a log‐linear effect of seabird‐derived nitrogen input on coral growth rates (Figure 2a; Appendix S5), and reef fish biomass increased as seabird‐derived nitrogen inputs increased (Figure 2b; Appendix S5). Reef functioning also increased with seabird‐derived nitrogen input; there was a log‐linear relationship between seabird‐derived nitrogen input and both parrotfish grazing (Figure 2c; Appendix S5) and bioerosion (Figure 2d; Appendix S5). If rats were predicted to be eradicated and 75% native vegetation cover was restored throughout the Chagos Archipelago, the shallow coral reef area of the Archipelago could support a 52% increase in reef fish biomass, resulting in 50,000 tons more reef fish (95% HPD: 7000–310,000).

## DISCUSSION

### Influence of island restoration on seabird populations

Congruent with global trends (Jones et al., 2008) and previous work in the Chagos Archipelago (Benkwitt, Gunn, et al., 2021; Benkwitt et al., 2022; Graham et al., 2018), rat presence had a negative effect on seabird breeding abundances, whereas the proportion of native habitat cover and island size had positive effects. We predicted that if rats were eradicated from the currently rat‐infested islands of the Chagos Archipelago, populations of lesser noddies, sooty terns, and red‐footed boobies across these islands could increase 18‐fold (although our predicted estimates are associated with large uncertainty). Although we were only able to make predictions for the 3 most abundant seabird species that inhabit the Chagos Archipelago, we assumed that less numerous species, particularly small species and those that nest in burrows (Jones et al., 2008), could likely experience similar population increases under rat eradication scenarios. For example, populations of brown boobies *S. leucogaster*, of which there are currently less than a thousand individuals throughout the Chagos Archipelago, are slowly increasing across rat‐free islands (P. Carr, personal communication). This being said, although lesser noddies, sooty terns, and red‐footed boobies are common, widespread seabird species that might be able to passively recover following invasive species eradications and native vegetation restoration, the restoration or reintroduction of other seabird populations, particularly procellariform species, is likely to benefit from targeted methods such as translocations and social attraction techniques (Spatz et al., 2023).

Numerous factors influence the probabilities, rates, and densities of seabird recovery, including the behavioral and demographic traits of the native seabird assemblage, the proximity to source populations and human activities, and the time since eradication (Borrelle et al., 2018). Although some populations take decades to recover (Jones, 2010a), others have rebounded rapidly following successful eradication attempts, driven by immigration and enhanced recruitment (Brooke et al., 2018) as well as interspecific differences in the traits that drive these processes (Philippe‐Lesaffre et al., 2023). For example, when rodent predation on eggs and chicks was eliminated by a successful eradication at a tropical Pacific atoll, breeding noddy densities increased swiftly (Philippe‐Lesaffre et al., 2023). Red‐footed booby populations recovered more slowly, potentially because they are larger and less at risk from predation, and are instead more influenced by the availability of suitable breeding habitat (Philippe‐Lesaffre et al., 2023).

Breeding habitat availability is a key mediator of seabird population recovery (Borrelle et al., 2018). We found that atoll island size and native habitat cover had positive influences on the abundances of breeding lesser noddies, sooty terns, and red‐footed boobies across the Chagos Archipelago due to increased availability of suitable nesting sites and habitats. In the Chagos Archipelago, prioritizing the restoration of human‐introduced coconut palm plantations to native habitat has been encouraged (Carr et al., 2021). Removing or rewilding abandoned coconut palm plantations, by undertaking revegetation efforts to restore native forest, savannah, and mixed shrub (likely to occur naturally as successional growth) across the Chagos Archipelago, would be optimal for its tropical seabird populations (Carr et al., 2021). Here, native forest habitats are preferable to coconut palm due to their more complex, stable canopies (McCauley et al., 2012), and savanna habitats can also support high abundances of ground‐nesting seabirds (Carr et al., 2021).

### Prey requirements of seabird populations

Another key determinant of seabird colonization is the accessibility, predictability, and quality of marine foraging habitats (Borrelle et al., 2018). Although previous work has focused on the potential benefits of island restoration for seabirds (Benkwitt, Gunn, et al., 2021; Carr et al., 2021; Jones et al., 2016; Spatz et al., 2022), we linked another crucial aspect of their life‐history strategies in the context of a large (640,000 km^2^) marine protected area that surrounds the Chagos Archipelago, in which fishing is illegal (Hays et al., 2020). This protection is valuable in ensuring epipelagic forage fish prey availability as well as reducing the threat of fisheries bycatch (Le Corre et al., 2012). Furthermore, the productivity of prey biomass in the central Indian Ocean is high due to the presence of highly productive taxa such as cephalopods, which have accelerated productivity in high‐temperature tropical waters (Rosa et al., 2019). Seabirds are huge consumers of marine biomass globally (Brooke, 2004; Cury et al., 2011), and we estimated that the Chagos Archipelago's entire current seabird population consumed a mean of 8300 tons of prey annually from the pelagic environment (with high associated uncertainty, largely due to the propagation of uncertainty around the restored seabird abundances). Despite high consumption levels, our size‐based model estimates suggested that there is generally adequate epipelagic prey available in the offshore waters around the Chagos Archipelago to sustainably support the energy requirements of restored lesser noddy, sooty tern, and red‐footed booby populations following rat eradication and native vegetation restoration measures. Although prey availability is more limited in the mean maximum foraging ranges of the seabirds (Appendix S6), birds from larger colonies often extend their foraging ranges to secure adequate resources (Ashmole, 1963; Trevail & Wood et al., 2023). In marine areas that do not have the same levels of protection as the Chagos Archipelago, conservation initiatives should consider the availability of forage fish prey alongside the availability of suitable breeding habitats, and interventions to limit key fisheries may need to be considered.

It is also important to consider the other consumers of these prey species (including tunas, dolphins, and sharks) that interact both competitively and facilitatively with seabirds (Pitman, 1986). For example, populations of over 570,000 grey reef sharks (*Carcharhinus amblyrhynchos*) and 31,000 silvertip sharks (*Carcharhinus albimarginatus*) (Ferretti et al., 2018) forage across both the reef and pelagic habitats of the Chagos Archipelago Marine Protected Area (Williamson et al., 2020). The prey requirements of these shark populations are likely to be an order of magnitude higher than those of the Archipelago's seabirds (grey reef sharks alone likely consume over 160,000 tons/year if they consume resources at a similar rate to those from Palmyra Atoll, Pacific Ocean [Dunn et al., 2022]). The marine predator assemblage is composed of a range of different niches, however, with grey reef sharks foraging closer to reef habitats and on larger bodied fish than many of the seabird species (Tickler et al., 2017). Indeed, inter‐taxa competition pressures will likely be negligible in the face of the restoration of entire terrestrial and marine ecosystems. This being said, although forage fish stocks are rarely targeted by industrial fishing in the Indian Ocean, in less protected areas there is high fishing pressure on taxa like tuna that seabirds facultatively forage with, which may in turn decrease the catchability of prey by seabirds (Danckwerts et al., 2014).

### Influence of restored seabird populations on coral reefs

In addition to exerting a large influence on offshore marine ecosystems via prey extraction, seabirds are ecosystem engineers in and near their breeding habitats, altering physical and chemical conditions (Grant et al., 2022). For example, fertile seabird guano enters adjacent nearshore marine ecosystems via, for example, direct defecation and groundwater discharge, and, although anthropogenic nutrients from agriculture and waste can increase the susceptibility of corals to bleaching, seabird‐derived nutrients deliver ratios of nitrogen and phosphorous that are beneficial to coral physiology (Wiedenmann et al., 2023). Indeed, seabird guano nutrient inputs enhance coral growth and recovery from bleaching events (Benkwitt et al., 2023).

Assuming that seabird population restoration is successful and that increased seabird‐derived nutrients enter nearshore systems, restored nutrient fluxes could realize a mean 52% increase in reef fish biomass throughout the Chagos Archipelago. Predicted increases in reef fish biomasses are due to seabird‐vectorized nutrients subsidizing coral reef ecosystems, propagating through the food web, and causing higher fish growth rates (Benkwitt, Taylor, et al., 2021; Graham et al., 2018). Increased reef fish biomasses adjacent to seabird‐dominated islands are particularly evident in herbivorous species, such as damselfish (turf algae consumers) and parrotfish, that target cyanobacteria and other autotrophic microorganisms (Benkwitt, Taylor, et al., 2021; Graham et al., 2018). Due to their novel feeding biology, parrotfish are an important group of fishes that perform unique ecosystem functions with their jaws, including the scraping and grazing of substrate and the bioerosion of dead corals (Clements et al., 2017). Rates of these functions, predicted to increase by 140% and 270% for grazing and bioerosion, respectively, on reefs adjacent to restored islands, are critical to reef recovery following disturbance events (e.g., storms and bleaching) because they help provide stable and clear substratum for new coral settlement, growth, and recovery (Benkwitt, Taylor, et al., 2021). The functional role of parrotfish is therefore likely to be increasingly important as coral bleaching events are predicted to increase in frequency with anthropogenic climate warming (Hughes et al., 2018). Furthermore, seabird nutrient‐driven increases in coral growth rates (mean increase = 90%) might help mitigate reefs against the threat of sea‐level rise and submergence (Perry et al., 2018).

### Significance and implications

Tropical marine ecosystems host areas of significant importance for seabirds and coral reefs, 2 of the most threatened marine communities on earth due to a combination of stressors including sea‐level rise and extreme temperature events. Our results from the Chagos Archipelago suggest that if invasive species were eradicated and native vegetation cover was restored, not only would large populations of seabirds have the potential to recover, but the oceanic prey base would also support recovered seabird populations. Although our results are subject to large uncertainty, our modeling of potential seabird recovery and cross‐ecosystem nutrient subsidy effects provides a new layer of information to consider when prioritizing island restoration efforts throughout the tropical Indian, Pacific, and Atlantic Oceans that seek to maximize the benefits of island restoration for both land and sea (Sandin et al., 2022). We encourage the consideration of such benefits in case‐by‐case island restoration planning that considers the broader ecological context and other island conservation prioritization frameworks (Holmes et al., 2019), outstanding protected area designations, and the land‐use requirements and cultural values held by any Indigenous peoples present. The results of such efforts can feed into future modeling exercises, reducing some of the uncertainty exhibited in this study.

Our study supports an emergent body of evidence suggesting that the delivery of elevated nutrients to nearshore coral reefs, via guano from adjacent seabird colonies, provides a bottom‐up nutrient subsidy that benefits coral reef ecology, thereby highlighting the need for ecosystem connectivity to be an explicit conservation priority. Investing in island habitat restoration and invasive species eradications provides tangible opportunities to revive lost seabird populations, reconnect broken nutrient pathways, and stem biodiversity losses across the globe's tropical atolls (Jones et al., 2016). Actions should be prioritized to reverse seabird population declines and rebuild an interconnected world in which nutrient cycling across the globe's lands and oceans is restored and accelerated (Doughty et al., 2016; Lundberg & Moberg, 2003).

## AUTHOR CONTRIBUTIONS

**Ruth Dunn**: Conceptualization; data curation; formal analysis; investigation; methodology; project administration; validation; visualization; writing—original draft. **Cassandra Benkwitt**: Conceptualization; investigation; resources; supervision; writing—review and editing. **Olivier Maury**: Resources; software; writing—review and editing. **Nicolas Barrier**: Resources; software; writing—review and editing. **Peter Carr**: Resources; writing—review and editing. **Nicholas Graham**: Conceptualization; funding acquisition; investigation; resources; supervision; writing—review and editing.

## Supporting information

Supporting Information
