# Supplementary material for: Island restoration to rebuild seabird populations and amplify coral reef functioning
Source: Conserv Biol. 2024 Jun 18;39(1):e14313. doi: 10.1111/cobi.14313 (PMC11780216; doi:10.1111/cobi.14313)
Supplement: Supplementary file 1 — Supporting Information [file COBI-39-e14313-s001.docx]

**Supporting Information**

Island restoration to rebuild seabird populations and amplify coral reef functioning

Additional supporting information may be found in the online version of the article at the publisher’s website.

Appendix S1. The 25 atoll islands of the Chagos Archipelago (central Indian Ocean) included within this study, alongside their area, rat status, the proportion of native vegetation cover, and the number of pairs of breeding red footed boobies, sooty terns and lesser noddies present.

| Atoll | Island | Rat status | Island area (ha) | Native vegetation cover (%) | Red footed boobies (pairs) | Sooty terns (pairs) | Lesser noddies (pairs) |
| --- | --- | --- | --- | --- | --- | --- | --- |
| Great Chagos Bank | Danger | Absent | 66 | 72 | 3,500 | 0 | 2,400 |
| Great Chagos Bank | Sea Cow | Absent | 18 | 66 | 1,400 | 0 | 1,200 |
| Great Chagos Bank | Eagle Island | Present | 243.5 | 28 | 10 | 0 | 10 |
| Great Chagos Bank | Nelson's Island | Absent | 81 | 88 | 3,300 | 0 | 12,000 |
| Peros Banhos | Coin | Present | 127.5 | 6 | 0 | 0 | 10 |
| Peros Banhos | Passe | Present | 22.5 | 8 | 54 | 0 | 40 |
| Peros Banhos | Moresby | Present | 43.5 | 35 | 540 | 0 | 105 |
| Peros Banhos | Parasol | Absent | 7.5 | 82 | 96 | 37,500 | 140 |
| Peros Banhos | Longue | Absent | 25.5 | 60 | 490 | 48,000 | 1,100 |
| Peros Banhos | Grand Bois | Absent | 14.5 | 66 | 127 | 0 | 2,000 |
| Peros Banhos | Petite Bois | Absent | 8.5 | 66 | 120 | 2,000 | 14,000 |
| Peros Banhos | Yeye | Present | 58.5 | 27 | 0 | 0 | 55 |
| Peros Banhos | Petite Coquillage | Absent | 17 | 73 | 125 | 20,000 | 1,850 |
| Peros Banhos | Grand Coquillage | Absent | 21 | 46 | 750 | 38,000 | 1,500 |
| Peros Banhos | Coin de Mire | Absent | 0.5 | 100 | 0 | 0 | 0 |
| Peros Banhos | Fouquet | Present | 2 | 14 | 0 | 0 | 0 |
| Salomon Islands | Boddam | Present | 108.5 | 3 | 0 | 0 | 10 |
| Salomon Islands | Diable | Present | 0.5 | 30 | 0 | 0 | 0 |
| Salomon Islands | Anglaise | Present | 75.5 | 8 | 0 | 0 | 10 |
| Salomon Islands | Passe | Absent | 26 | 24 | 27 | 0 | 400 |
| Salomon Islands | Mapou | Absent | 4 | 33 | 100 | 0 | 1,600 |
| Salomon Islands | Takamaka | Present | 44 | 37 | 302 | 0 | 100 |
| Salomon Islands | Fouquet | Present | 39.5 | 26 | 17 | 0 | 0 |
| Salomon Islands | Sepulture | Present | 0.5 | 11 | 0 | 0 | 0 |
| Salomon Islands | Poule | Present | 0.5 | 74 | 0 | 0 | 0 |

Rat status and island area data: (Carr et al., 2021); Vegetation data (Wilkinson, 2017)**;** Seabird population data: (Carr et al., 2020)**.**


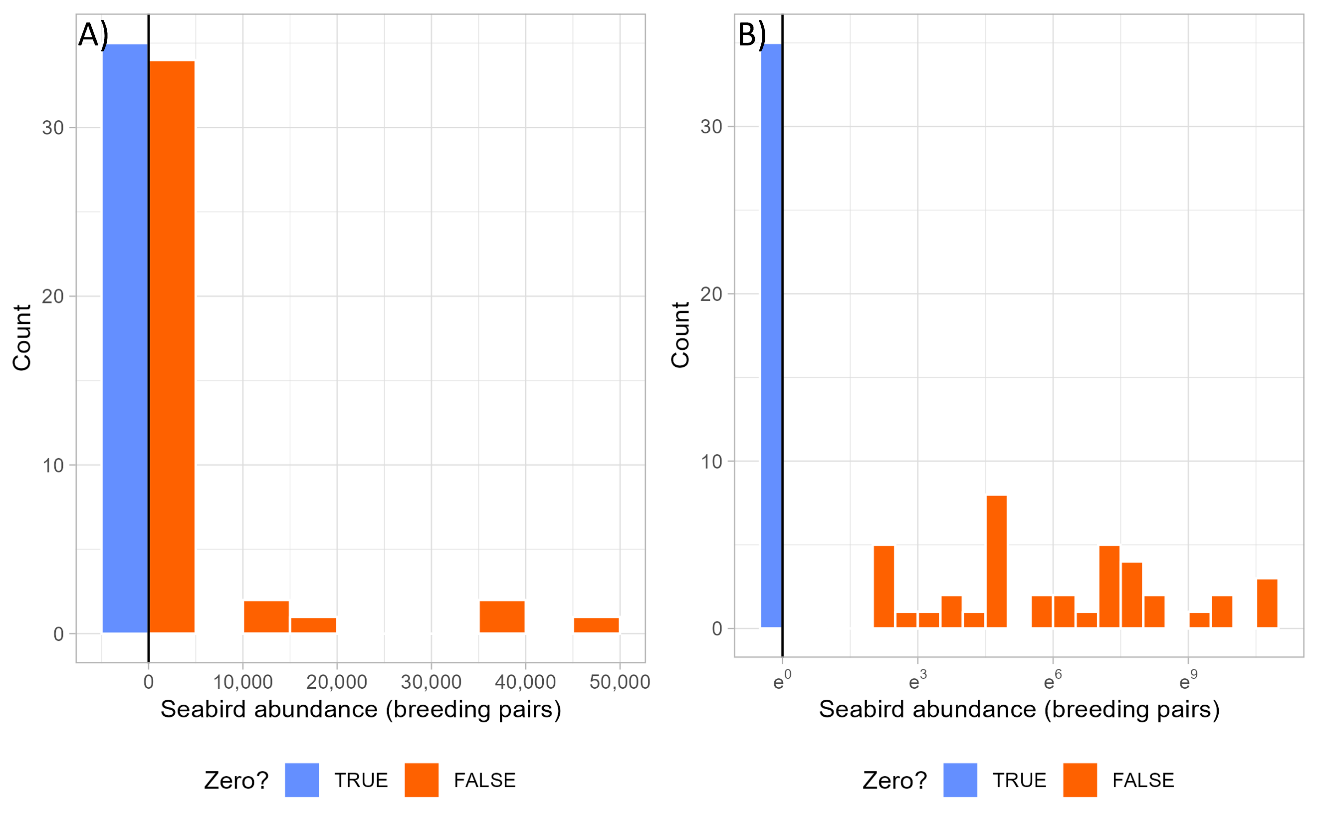


Appendix S2. Histograms of the Chagos Archipelago seabird abundance data, illustrating A) the near exponentially-shaped original data and B) the more normally-shaped logged data, as well as a high proportion of zero data.

Appendix S3. Prior distributions for the parameters used to model the influence of rat status, island area (ha), and the proportion of native vegetation cover on seabird abundances throughout the Chagos Archipelago. We used a Bayesian hurdle lognormal regression model that was a mixture of two processes, dependent on whether seabird abundance values were larger than zero, as influenced by rat status (‘present’ or ‘absent’).

| Variable | Explanation | Prior |
| --- | --- | --- |
| *mu* |  |  |
| Intercept: Red-footed booby *Sula sula* | Expected intercept of red-footed booby abundance | $N\left( 6, 1 \right)$ |
| Species: Sooty tern *Onychoprion fuscatus* | Expected difference in sooty tern numbers (in comparison to red-footed boobies) | $N\left( 1, 0.5 \right)$ |
| Species: Lesser noddy *Anous tenuirostris* | Expected difference in lesser noddy numbers (in comparison to red-footed boobies) | $N\left( 0, 0.5 \right)$ |
| *Rattus rattus* presence | Expected negative influence of rats | $N\left( -1, 0.5 \right)$ |
| log (island area) | Expected positive influence of island area | $N\left( 1, 0.5 \right)$ |
| Proportion of native vegetation cover | Expected positive influence of native vegetation cover | $N\left( 1, 0.5 \right)$ |
| *hu* |  |  |
| Intercept | As 46% of our data our 0s, we expect that ~40% of the 0s are caused by rat presence | $N\left( -0.4, 0.05 \right)$ |
| *Rattus rattus* presence | Expected positive influence of rats on the data being 0 | $N\left( 1, 0.5 \right)$ |
| *random effect* |  |  |
| Atoll, Island | Default prior | $t\left( 3, 0, 6.8 \right)$ |

**Appendix S4**. Posterior estimates for the effects of island habitat on seabird abundances (breeding pairs of lesser noddies, sooty terns and red-footed boobies) across 25 atoll islands within the Chagos Archipelago.

| Effect |  | 95% HPD | |
| --- | --- | --- | --- |
|  | Mean | 2.5 | 95.7 |
| *hu* |  |  |  |
| Intercept: Red-footed booby *Sula sula* | -1.13 | -1. 51 | -0.78 |
| *Rattus rattus* presence | 1.42 | 0.74 | 2.10 |
| *mu* |  |  |  |
| Intercept | 5.77 | 5.01 | 6.50 |
| *Rattus rattus* presence | -1.36 | -2.19 | -0.53 |
| Island area | 0.45 | -0.25 | 1.19 |
| Proportion of native vegetation cover | 1.25 | 0.68 | 1.84 |
| Species: Sooty tern *Onychoprion fuscatus* | 1.77 | 0.90 | 2.65 |
| Species: Lesser noddy *Anous tenuirostris* | 0.19 | -0.56 | 0.86 |
| *random effect* |  |  |  |
| Island | 0.48 | 0.01 | 1.06 |

Appendix S5. Posterior estimates for the effects of seabird-derived nitrogen inputs on coral reef metrics throughout the Chagos Archipelago.

| Models and effects | N _islands_ |  | 95% HPD | |
| --- | --- | --- | --- | --- |
|  |  | Mean | 2.5 | 95.7 |
| *Coral growth* |  |  |  |  |
| Intercept | 8 | 4.36 | 3.88 | 4.83 |
| Seabird-derived nitrogen input |  | 0.11 | -0.072 | 0.26 |
| Random effect: island |  | 0.59 | 0.0028 | 1.08 |
| *Reef fish biomass* |  |  |  |  |
| Intercept | 12 | 6.29 | 5.99 | 6.58 |
| Seabird-derived nitrogen input |  | 0.07 | -0.033 | 0.17 |
| Random effect: island |  | 0.41 | 0.045 | 0.77 |
| *Parrotfish grazing* |  |  |  |  |
| Intercept | 12 | 1.99 | 1.61 | 2.36 |
| Seabird-derived nitrogen input |  | 0.15 | 0.028 | 0.28 |
| Random effect: island |  | 0.37 | 0.00017 | 0.83 |
| *Parrotfish bioerosion* |  |  |  |  |
| Intercept | 12 | 3.83 | 3.19 | 4.49 |
| Seabird-derived nitrogen input |  | 0.215 | -0.01 | 0.45 |
| Random effect: island |  | 0.84 | 0.01 | 1.72 |


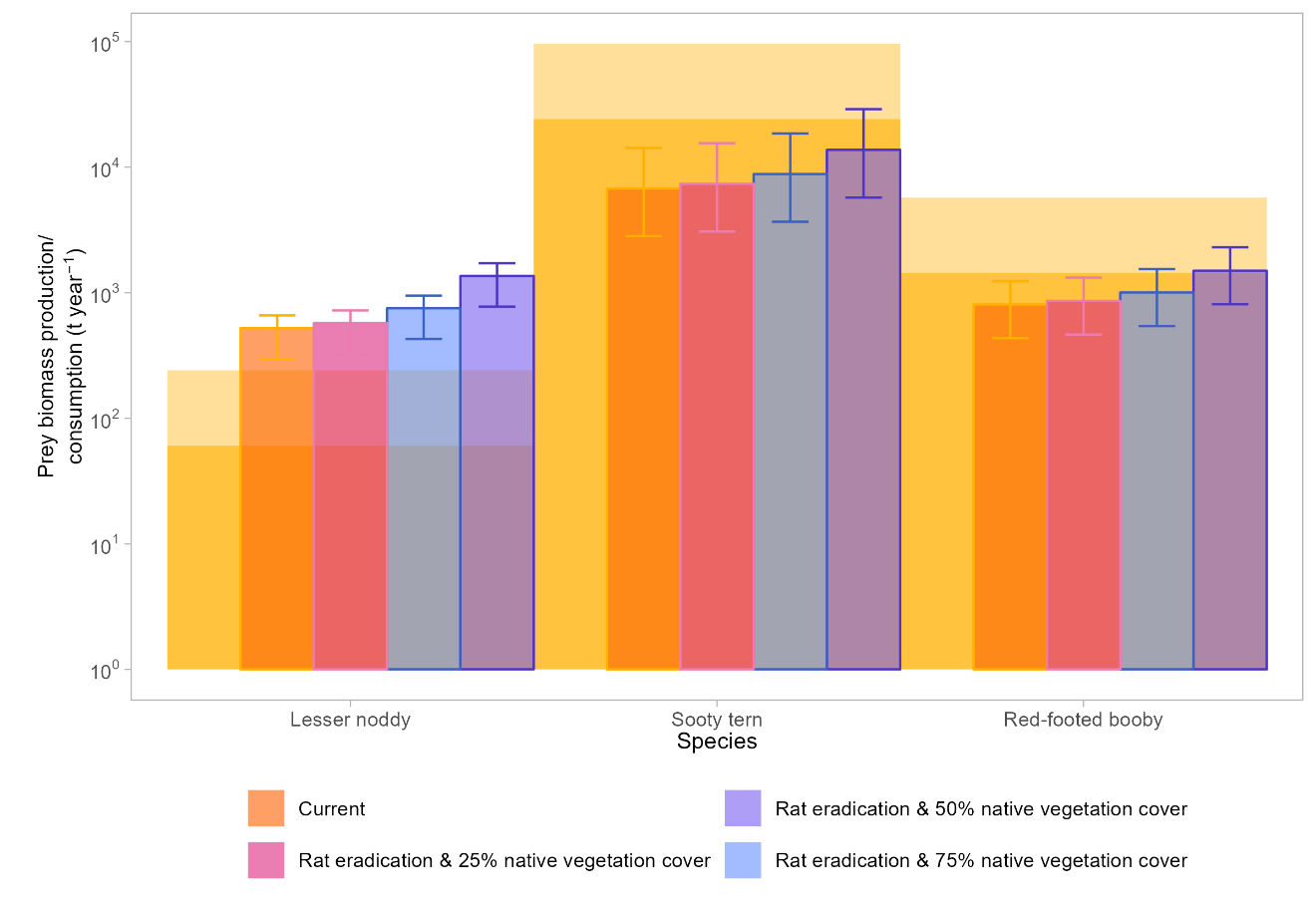


Appendix S6. Epipelagic prey biomass production (tonnes.year^-1^) within the mean maximum breeding foraging ranges of lesser noddies *Anous tenuirostris*, sooty terns *Onychoprion fuscatus*, and red-footed boobies *Sula sula* throughout the Chagos Archipelago (shown in yellow with darker and lighter shading indicating lower and higher ranges of biomass production respectively), in relation to the their consumption requirements (all on the log-scale), under current conditions (orange) as well as under three rat eradication and native habitat restoration scenarios (all on the log-scale). The height of the bars represents the posterior mean, and the error bars represent the upper and lower Bayesian credible intervals, as derived from the ‘Seabird FMR Calculator’ (Dunn et al. 2018).

**SI References**

Carr, P., Trevail, A., Bárrios, S., Clubbe, C., Freeman, R., Koldewey, H. J., Votier, S. C., Wilkinson, T., & Nicoll, M. A. C. (2021). Potential benefits to breeding seabirds of converting abandoned coconut plantations to native habitats after invasive predator eradication. *Restoration Ecology*, **29**, e13386.

Carr, P., Votier, S., Koldewey, H., Godley, B., Wood, H., & Nicoll, M. A. C. (2020). Status and phenology of breeding seabirds and a review of Important Bird and Biodiversity Areas in the British Indian Ocean Territory. *Bird Conservation International*, **31**, 14–34.

Wilkinson, T. (2017). *Vegetation maps of the Chagos Archipelago*. https://chip.chagos-trust.org/
